# Notes

**Published:** 1898-06-18

**Authors:** 


### The Institutional Workshop.

### NOTES.

The improvements which have been introduced in the arrangement for the attendance of the medical officers in the receiving room at The London Hospital are stated to work well. Patients are cleared off as soon as they arrive, and but little delay in their treatment occurs. Each of the medical officers in turn comes on duty at 8 a.m., and one or other of them is on duty until 11.30 p.m.

Up to June 1st the result of the appeal for the fifth Quinquennial Maintenance Fund of The London Hospital has been the collection of £4,141 in new annual subscriptions, £4,464 old annual subscriptions renewed, £39,700 in new donations, and £3,896 from donors to the previous funds. This is in addition to the £35,000 promised for the new out-patient department and the new operating theatre.

Not only have a large proportion of the aristocratic ladies now assembled in London promised their aid as stall holders at the Press Bazaar in aid of the London Hospital on June 28th and 29th, but a number of the leading members of the stage will lend their assistance also. Amongst them are Miss Ellen Terry, Sir Henry Irving, Mr. Beerbohm Tree, Mr. George Alexander, Mr. Cyril Maude, Miss Fay Davies, Miss Violet Vanbrugh, Miss Cissy Loftus, Miss Marie Tempest, Mr. Albert Chevalier, and many others. Two performances will be held each day, at three or half-past three and eight o'clock. Tickets for these may be obtained at Whiteley's and the usual agents.

